# What Makes a Neighborhood? Associations Between Behavioral and Psychosocial Factors and Perceptions of Neighborhood Environments Among Community-Dwelling Older Black and Latino Adults

**DOI:** 10.3390/ijerph23020196

**Published:** 2026-02-03

**Authors:** Crystal M. Glover, Ana W. Capuano, Tianhao Wang, Brittney S. Lange-Maia, David A. Bennett, David X. Marquez, Lisa L. Barnes, Julie A. Schneider, Melissa Lamar

**Affiliations:** 1 Department of Neurology, Gillespie Neuroscience Research Facility, School of Medicine, University of California, Irvine, CA 92697, USA; 2 Department of Neurology, University of Chicago, Chicago, IL 60637, USA; ana.capuano@bsd.uchicago.edu; 3 Department of Public Health, University of Chicago, Chicago, IL 60637, USA; 4 Rush Alzheimer’s Disease Center, Rush University Medical Center, Chicago, IL 60612, USA; tianhao_wang@rush.edu (T.W.); brittney_lange-maia@rush.edu (B.S.L.-M.); david_a_bennett@rush.edu (D.A.B.); marquezd@uic.edu (D.X.M.); lisa_l_barnes@rush.edu (L.L.B.); julie_a_schneider@rush.edu (J.A.S.); melissa_lamar@rush.edu (M.L.); 5 Department of Neurological Sciences, Rush Medical College, Chicago, IL 60612, USA; 6 Department of Family and Preventive Medicine, Rush Medical College, Chicago, IL 60612, USA; 7 Department of Kinesiology and Nutrition, University of Illinois Chicago, Chicago, IL 60607, USA; 8 Department of Psychiatry and Behavioral Sciences, Rush Medical College, Chicago, IL 60612, USA; 9 Department of Pathology, Rush Medical College, Chicago, IL 60612, USA

**Keywords:** behavioral factors, psychosocial factors, perceptions of neighborhood environments, individual determinants, Black adults, Latinos

## Abstract

**Highlights:**

**Public health relevance—How does this work relate to a public health issue?**

- How people perceive their neighborhood environments can impact their aging trajectories, independent of objective measures.
- Previous studies largely do not emphasize how non-Latino Black and Latino older adults perceive their neighborhood environments and the impact of behavioral and psychosocial factors.

**Public health significance—Why is this work of significance to public health?**

- Non-Latino Black and Latino older adults reported overall neutral perceptions of their neighborhoods.
- Better neighborhood perceptions were associated with less discrimination and higher income for non-Latino Black older adults, while more purpose in life was related to better neighborhood perceptions among older Latinos.

**Public health implications—What are the key implications or messages for practitioners, policy makers and/or researchers in public health?**

- Intervention strategies must exist at the individual, social, and structural levels and focus on income, discrimination, physical activity, late life social activity, and purpose in life among non-Latino Black and Latino older adults.
- When combined, these multilevel strategic efforts may benefit structural and social characteristics of neighborhood environments as well as neighborhood perceptions which, in turn, can facilitate overall health and quality of life for non-Latino Black and Latino older adults.

**Abstract:**

How people perceive their neighborhoods can impact their aging trajectories, with less known regarding neighborhood perceptions among older adults from minoritized groups. This study examined the impacts of behavioral and psychosocial factors on neighborhood perceptions among non-Latino (NL) Black and Latino older adults. Participants (N = 506) were NL Black (n = 372) and Latino (n = 134) older adults ($\bar{x}$ age = 79 years) without dementia. Participants completed a modified Perceptions of Neighborhood Environments Scale (mPNES; higher scores indicate more favorable perceptions) and measures of behavioral and psychosocial factors. We performed fully saturated linear regression analyses to assess how each factor related to the mPNES, followed by stepwise linear regression analyses to determine final predictive models for the full sample and each ethnoracial group. For the full sample, higher purpose in life, more physical activity, less discrimination, and higher income were associated with higher mPNES scores. For NL Black older adults, more physical activity, less discrimination, and higher income were associated with higher mPNES scores. For older Latinos, more purpose in life and a larger social network size were associated with higher mPNES scores. Distinct associations exist by ethnoracial group and suggest unique considerations to facilitate positive neighborhood perceptions among NL Black and Latino older adults.

## 1. Introduction

Neighborhood environments play a critical role in the health and wellbeing of older adults, especially structural characteristics or designed features [1,2,3,4]. Positive structural characteristics provide a neighborhood and its residents with maintained walkways, the availability of safe parks and trails, access to reliable modes of transportation, and proximity to healthcare facilities, locations for physical and social activities, and fresh fruits and vegetables. Older adults who reside in these structurally well-characterized neighborhoods experience better mobility, lower likelihood of early mortality, reduced levels of cognitive impairment, and a decreased risk of dementia [4,5,6,7]. Unfortunately, older adults who belong to minoritized racial and ethnic groups, including non-Latino (NL) Black and Latino older adults, oftentimes do not live in neighborhood environments that support their health, placing these communities at an elevated risk for deleterious outcomes including cognitive impairment, mobility limitations, and poorer mental health [1,3,8].

Structural neighborhood characteristics are not the sole contributor to health in aging [4]. Positive social elements of neighborhood environments, such as friendly and respectful relationships between neighbors and a sense of belonging, also support healthy aging. Older adults who reside in neighborhoods marked by more optimal social attributes report better mental health, higher levels of wellbeing, and feelings of being supported [5]. Structural and social characteristics of neighborhood environments, individually and collectively, remain highly important for older adults and their health. Yet, they do not fully capture how a person perceives their neighborhood environment, which can also impact their health in aging trajectories—independent of objective measures, particularly for members of minoritized groups [9,10,11,12].

Perceptions of neighborhood environments refer to how people make sense of or interpret the characteristics of where they live—independent of objective measures or the viewpoints of others [9,13]. More favorable neighborhood perceptions regarding walkability, local parks, grocery stores, and physical appearance have been linked to more physical activity [6,12], higher levels of wellbeing [14], and better self-rated physical and mental health [15,16]. Conversely, unfavorable neighborhood perceptions have been associated with lower cognitive function [17]. Previous research has also suggested that NL Black and Latino adults possess more negative perceptions of their neighborhood environments compared to their NL White counterparts [18,19]. However, studies pertaining to neighborhood perceptions have been conducted with samples largely comprising either NL White adults or persons younger than 65 years of age, without much emphasis on how *NL Black and Latino older adults* perceive their neighborhood environments and why.

**Figure A1:**
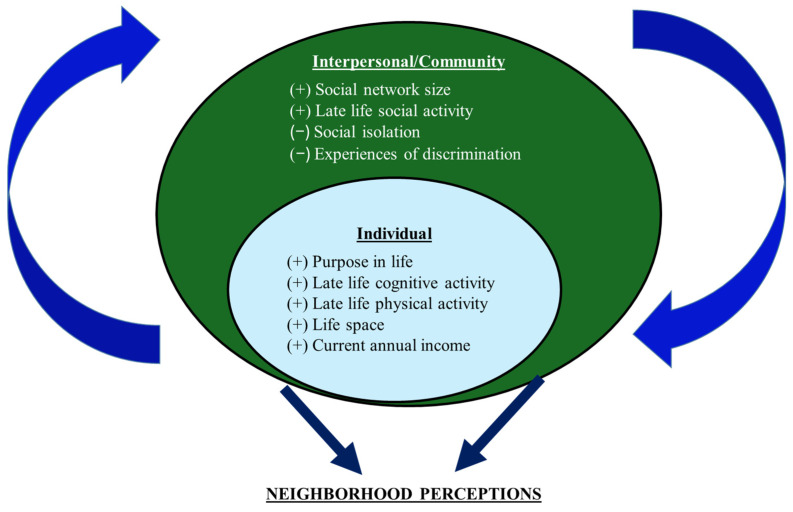
Hypothesized Relationships Between Individual- and Interpersonal-Level Factors and More Favorable Perceptions of Neighborhood Environments.

The current study: (1) characterized how NL Black and Latino older adults perceived their neighborhood environments and (2) examined the impacts of behavioral and psychosocial factors on neighborhood perceptions among these older adults. We defined neighborhoods as the area within a 20 min walk from a person’s house. We included both NL Black and Latino older adults in the current examination as they face an increased risk of deleterious health outcomes in aging. Suboptimal aging among NL Black and Latino older adults in the United States is, in part, influenced by discrimination and related practices such as redlining and the facilitation of economic disparities. Equally, NL Black and Latino older adults exhibit resilience and coping factors such as familial support, cultural practices and celebrations, and faith-based belief systems. While NL Black and Latino older adults undoubtably share lived experiences, they also have their own historical and current distinctions related to migration, immigration, and other circumstances. Cumulatively, these issues potentially inform behavioral and psychosocial factors—both common and unique—at the intersection of the individual and their surroundings that may impact neighborhood perceptions. The Ecological Theory of Aging [20,21] and the World Health Organization (WHO) Aging-Friendly Cities and Communities Framework [22] guided the selection of our behavioral and psychosocial factors among existing data at the Rush Alzheimer’s Disease Center (RADC). Behavioral factors included late life cognitive activity, late life social activity, and physical activity—individual-level variables associated with older adults’ participation in their communities, a central goal of the WHO Aging-Friendly Cities and Communities Framework [22]. Psychosocial variables consisted of purpose in life, social isolation, social network size, life space, experiences of discrimination, and current annual income and represented individual- and interpersonal-level factors that may illuminate the relationship between an older adult and their neighborhood environment, a core tenet of the Ecological Theory of Aging [9,20,21]. We postulate, as informed by the Ecological Theory of Aging, that these behavioral and psychosocial factors may be associated with neighborhood perceptions through the person–environment fit or how well someone is supported within their residential area via aspects of the built environment, nearby resources, and community relationships. We hypothesize that greater levels of cognitive, social, and physical activities in late life, purpose in life, social network size, life space, and current annual income will be associated with better neighborhood perceptions. Conversely, we hypothesize that lower levels of social isolation and experiences of discrimination will be related to better neighborhood perceptions. This study ultimately aimed to understand neighborhood perceptions among NL Black and Latino older adults that may point to targets for policy development, public health intervention, and community support that can facilitate equitable environmental outcomes in aging for all [22]. See Figure A1 for hypothesized relationships between behavioral and psychosocial factors and neighborhood perceptions.

## 2. Materials and Methods

### 2.1. Participants

Eligible participants for the current analyses self-identified as either NL Black or Latino and were participating in one of four ongoing cohort studies on aging and cognitive health at the RADC including the Rush Memory and Aging Project (MAP) [23], the Minority Aging Research Study (MARS) [24], the Rush Clinical Core, and the Rush Latino Core [25]. Each cohort study recruits from communities in the Chicago metropolitan area. All cohort participants are approximately 60 years of age or older, without known dementia, and tested within their homes. All cohort participants report their race (e.g., Black/African American) and ethnicity (i.e., Hispanic: yes or no) based on categories from the 1990 United States Census Bureau as well as their sex (i.e., male or female), date of birth, and years of education. As part of their cohort study participation, each person consents to an annual clinical evaluation that includes assessments of behavioral, biological, environmental, psychosocial, and sociocultural factors [23,24,25].

A substudy on characteristics and perceptions of neighborhood environments was introduced into the four cohort studies in 2020. Of the 1653 participants who self-identified as either NL Black or Latino with a complete baseline on or before the time of the current analyses (August 2022), 556 died before taking part in the neighborhood environments substudy. Of the remaining 1097 participants, 539 (49%) completed the substudy and 558 (51%) did not complete the substudy. Of the 539 who completed the substudy, 31 were diagnosed with dementia at our analytic baseline (first modified Perceptions of Neighborhood Environments Scale (mPNES) data point) and 2 had missing dementia diagnoses. As such, 506 participants were included in the current analyses. To assess potential selection bias, we compared eligible participants who completed the substudy to those who did not on key demographic variables including age, current annual income, ethnoracial group, and cognition (i.e., MMSE score). We noted differences in age (*p* = 0.02) and MMSE scores (*p* ≤ 0.001) with current study participants being younger (mean age = 71 years) with higher MMSE scores (mean = 28) compared to non-completers (mean age = 72 years and mean MMSE score = 27). We noted no differences in income (*p* = 0.10) and ethnoracial group (*p* = 0.41).

All cohort studies and the substudy on neighborhood environments were approved by an Institutional Review Board at Rush University Medical Center. Each participant signed an informed consent document for each study. Data can be requested at https://www.radc.rush.edu (accessed on 30 January 2026).

### 2.2. Outcome Variable: Modified Perceptions of Neighborhood Environments Scale (mPNES)

We used a modified version of the PNES to measure participants’ thoughts and experiences related to where they live [10,11,26,27]. We reduced the original 36-item measure to 12 items to lessen participant burden; however, we ensured that all original neighborhood dimensions were assessed including aesthetic quality, social features, physical activity spaces, and food availability. Instructions for the mPNES defined a neighborhood as “the area within about a 20-min walk (or about a mile) from your house.” The following instructions were also provided to participants: participants were asked to rate items along a Likert-type scale from 1 (strongly agree) to 5 (strongly disagree). All mPNES items, some of which were reverse coded so that a higher score reflected more positive or favorable neighborhood perceptions, were averaged to create an mPNES total score. The Cronbach’s α for the total mPNES was 0.79.

We also ran a principal component analysis (PCA) with varimax rotation to determine appropriate subscales for all RADC participants using their mPNES data (N = 964). Three components with eigenvalues greater than 1.0 were retained based on the scree plot, collectively explaining 63% of the total variance. The resulting PCA-derived factor loadings reflected the following constructs: (1) community cohesiveness (6 items) with a Cronbach’s α of 0.86; (2) health opportunities (4 items) with a Cronbach’s α of 0.74; and (3) ambient surroundings (2 items) with a Cronbach’s α of 0.57. We note a somewhat lower internal consistency reliability for the ambient surroundings subscale, which may reflect the small number of items or heterogeneity of environmental exposures captured by these constructs.

The community cohesiveness subscale referred to a person’s relationship with their neighbors and aesthetic conditions of their neighborhood. The health opportunities subscale denoted how the neighborhood supports participants’ options for selecting healthier food and taking part in physical activities. The ambient surroundings subscale assessed noise and traffic associated with the neighborhood environment. See Table A1.

### 2.3. Behavioral and Psychosocial Factors

Guided by the Ecological Theory of Aging [20,21] and the WHO Aging-Friendly Cities and Communities Framework [22], we included behavioral and psychosocial variables available at the RADC to understand their associations with neighborhood perceptions. Behavioral factors included late life cognitive activity, late life social activity, and physical activity. Psychosocial factors included purpose in life, social isolation, social network size, life space, experiences of discrimination, and current annual income.

### 2.4. Behavioral Factors

For late life cognitive activity, participants self-reported their engagement in seven activities (e.g., reading magazines or books) during the past year using a 5-point Likert scale (1 = every day/almost every day to 5 = once a year or less) [28,29]. Scores were reverse-coded and averaged with higher scores indicating more late life cognitive activity. Late life social activity was measured with six items assessing the frequency of participation in events such as going on day or overnight trips on a 5-point Likert scale (1 = once a year or less to 5 = every day or almost every day) [30]. Items were averaged with higher scores signaling higher levels of late life social activity. For physical activity, participants indicated whether they engaged in three activities (e.g., gardening or yardwork) within the past two weeks. If so, participants reported the number of occasions for each activity [29].

### 2.5. Psychosocial Factors

Purpose in life was measured using a 10-item instrument stemming from Ryff’s Scales of Psychological Well-Being [31,32]. Items assessed participants’ ability to derive meaning from life experiences and being goal-directed (e.g., “Some people wander aimlessly through life, but I am not one of them.”) using a 5-point Likert scale (1 = strongly disagree to 5 = strongly agree). Items were averaged for a total score with higher scores indicating more purpose in life. Social isolation referred to feeling detached from others and was measured with the modified Loneliness Scale consisting of five items (e.g., “I feel like I don’t have enough friends.”) along a 5-point Likert scale (1 = strongly disagree to 5 = strongly agree) [33]. All items were averaged to create an overall score with higher scores indicating more social isolation. Relatedly, social network size was a count of the number of children, family, and friends that a participant saw at least once a month [34]. Life space referred to a person’s spatial movement throughout their environment and was measured using a modified Life Space Questionnaire [35]. Participants responded “yes” or “no” to six items related to movement in six specific spatial zones (e.g., outside of one’s neighborhood represents a zone) in the past week. Item responses were summed with higher scores indicating a less constricted or larger life space. Experiences of discrimination referred to a participant’s reported encounters of being treated unfairly in everyday situations. Nine items (e.g., “You received poorer service than other people at restaurants or store.”) were framed in a general context without mention of race, gender, or age and rated along a 4-point Likert scale (1 = often to 4 = never). Responses were recoded to a binary format, with response options of often and sometimes = 1 and response options of rarely and never = 0, then summed across items to create a total score (range = 0–9), with higher scores indicating more frequent experiences of discrimination [36,37]. Income level was measured using the Show-Card Method from the Established Populations for Epidemiologic Studies of the Elderly, with participants asked to select 1 ($0.00 to $4999) of 10 ($75,000 and over) levels of total annual family income [38].

### 2.6. Analyses

To characterize how NL Black and Latino older adults perceive their neighborhood environments, we ran basic descriptive analyses, including mean, standard deviation, and range of scores, for the mPNES total scale and subscales across the full sample and by ethnoracial group (NL Black and Latino). To examine initial relationships between behavioral and psychosocial factors (predictor variables) and mPNES scores (separate outcomes for the total scale and each subscale), we performed fully saturated models (all predictor variables included) using linear regression analyses with the total sample and stratified by ethnoracial group. We performed subscale analyses to understand potential nuances in neighborhood perceptions. We stratified the sample by ethnoracial group to assess unique within-group relationships between predictor and outcome variables.

We established a final set of predictor variables associated with total mPNES scores and each subscale using separate stepwise linear regression models using the forward selection and backward elimination method for the full sample and stratified by ethnoracial group. For each predictor variable, we considered the statistical significance of entering and retention in the model as 0.10 and 0.05, respectively. All final models consisted of predictors with a statistical significance of *p* < 0.05. All models adjusted for age, gender, and years of education.

Lastly, we calculated the adjusted R-squared statistic for each model to compare fully saturated models with their respective final models as the models varied in the number of predictor variables. All analyses were conducted using SAS software, version 9.4 of the SAS system for Linux.

## 3. Results

### 3.1. Participant Characteristics

Participants (N = 506) were self-identified NL Black (n = 372) and Latino (n = 134) older adults with a mean age of 79 years and 14 mean years of education. Participants were 84% women and had a median MMSE score of 28. See Table A2 for additional participant characteristics.

### 3.2. Perceptions of Neighborhood Environments

Across the full sample, the total mPNES score was 47.69, with NL Black older adults ($\bar{x}$ = 49.65) reporting higher mPNES total scores than Latino older adults ($\bar{x}$ = 42.26). For both NL Black and Latino older adults, the Community Cohesiveness subscale of the mPNES received the highest scores. See Table A2.

**Figure A2:**
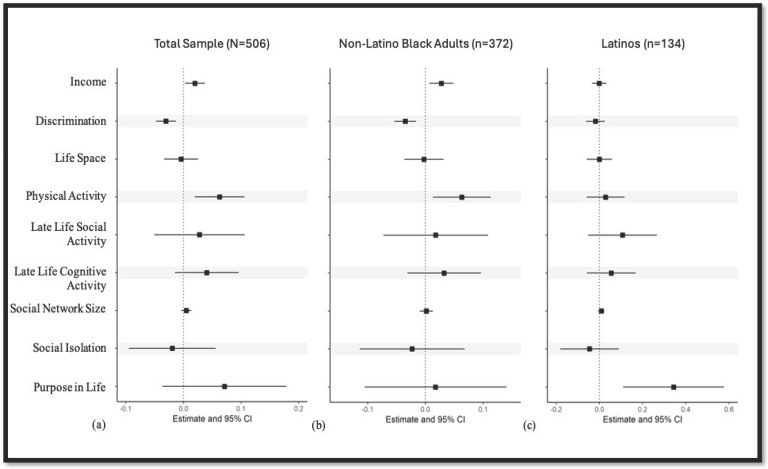
Forest Plots of the Fully Saturated Model Estimates and 95% Confidence Intervals (CI) for the Linear Regression Analyses Investigating the Relationship Between Behavioral and Psychosocial Factors and the Modified Perception of Neighborhood Environment Scale Total Score for (**a**) The Total Sample and Stratified By (**b**) Non-Latino Black Older Adults and (**c**) Latino Older Adults, All Adjusting for Age, Gender, and Years of Education.

We note smaller numbers of older Latinos (n = 134) compared to NL Black older adults (n = 372) and we emphasize interpretation of study results based on confidence intervals (see Figure A2) and stratified findings.

Lastly, we calculated the adjusted R-squared statistic for each model. Adjusted R-squared statistics either remained the same from fully saturated to final models or fluctuated by 0.01. See Table A3, Table A4, Table A5 and Table A6 for R-squared statistics for each model.

### 3.3. Associations Between Behavioral and Psychosocial Factors and Neighborhood Perceptions

#### 3.3.1. mPNES Total Scale

The initial saturated model included associations between more physical activity, less discrimination, and higher income with more positive neighborhood perceptions among the full sample and for NL Black older adults. For older Latinos, higher purpose in life was associated with more favorable neighborhood perceptions.

More physical activity, less discrimination, and higher income persisted from the initial saturated model into the final stepwise model for the full sample, with more purpose in life reaching the threshold for significance in the final model only. The final stepwise model with NL Black older adults included more physical activity, less discrimination, and higher income—all predictors in the initial saturated model. More purpose in life persisted from the initial saturated model into the final stepwise model for older Latinos, and larger social network size was introduced in the final model. See Table A3 and Figure A2 for more details regarding fully saturated models investigating relationships between behavioral and psychosocial factors and the mPNES.

#### 3.3.2. mPNES Community Cohesiveness Subscale

The initial saturated model for the full sample included higher purpose in life, more physical activity, and less discrimination associated with more favorable perceptions of community cohesiveness. For NL Black older adults, the initial saturated model consisted of associations between more physical activity, less discrimination, and higher income with more positive perceptions of community cohesiveness. For older Latinos, the initial saturated model included a relationship between higher purpose in life and more positive perceptions of community cohesiveness.

More purpose in life, more physical activity, and less discrimination persisted from the initial saturated model into the final stepwise model for the full sample, with higher income reaching the threshold for significance in the final model. More physical activity, less discrimination, and higher income remained in the final stepwise model from the initial saturated model for NL Black older adults, and less social isolation became statistically significant in the final model. The final stepwise model with older Latinos included higher purpose in life, like the initial saturated model. See Table A4.

#### 3.3.3. mPNES Health Opportunities Subscale

The initial saturated model did not include relationships between any behavioral and psychosocial factors and health-specific neighborhood perceptions for the full sample or for older Latinos. Conversely, less discrimination and higher income were associated with more favorable health-specific neighborhood perceptions among NL Black older adults.

The final stepwise model for the full sample included more physical activity and higher income, dissimilar to the initial saturated model. For NL Black older adults, the final stepwise model included less discrimination and higher income, like the initial saturated model. For older Latinos, the final stepwise model included more late life social activity, unlike the initial saturated model. See Table A5.

#### 3.3.4. mPNES Ambient Surroundings Subscale

The initial saturated model with the full sample included associations between higher purpose in life, smaller life space, less discrimination, and higher income and more positive ambient-specific perceptions. For NL Black older adults, the initial saturated model set forth relationships between smaller life space, less discrimination, and higher income with more favorable ambient-specific perceptions. For older Latinos, the initial saturated model included an association between higher purpose in life and more favorable ambient-specific perceptions.

All associations persisted from the initial saturated model into the final stepwise model for the full sample. In the final model for NL Black older adults, higher income remained, while smaller life space and less discrimination did not. For older Latinos, higher purpose in life remained in the final stepwise model, like the initial saturated model, and higher income became statistically significant. See Table A6.

## 4. Discussion

The current study examined behavioral and psychosocial factors associated with neighborhood perceptions among community-dwelling NL Black and Latino older adults without dementia. Neighborhood perceptions consisted of three components: community cohesiveness, health opportunities, and ambient surroundings. Multiple behavioral and psychosocial factors were considered based on the Ecological Theory of Aging [20,21] and the WHO Aging-Friendly Cities and Communities Framework [22]. Overall, participants held neutral opinions of their neighborhoods, with community cohesiveness rated the highest subscale among both NL Black and Latino older adults. Associations existed between behavioral and psychosocial factors and neighborhood perceptions, for all participants as well as specific to NL Black and Latino older adults, respectively. We observed persistent associations with more favorable neighborhood perceptions by ethnoracial group, including higher income for NL Black older adults and higher purpose in life for older Latinos. These results can lay the foundation for strategies to maintain or improve neighborhood perceptions among NL Black and Latino older adults that may, in turn, foster positive health outcomes in aging.

The current study adds to the growing body of literature that pertains to neighborhood perceptions among older adults [39,40]. Findings from the full sample include associations of higher levels of physical activity and income and lower levels of discrimination with better overall neighborhood perceptions. Additionally, higher income was linked to more positive perceptions of community cohesiveness and health- and ambient-related aspects of neighborhood environments for the full sample. A well-established relationship exists between higher income levels and structurally well-characterized neighborhoods, which may lend itself to better neighborhood perceptions [41,42]. Similarly, discrimination represents a key consideration in neighborhood perceptions, with less discrimination signaling more positive social aspects of a neighborhood, including residents feeling welcomed and comfortable among neighbors and local businesses [43,44]. A recent synthesis also suggests that more physical activity is indicative of more optimal objective structural and social aspects of a neighborhood environment, one typified by access to quality and safe spaces to partake in exercise and other movement [45,46]. Perhaps a more nuanced iterative relationship exists between better neighborhood perceptions and necessary, pleasing, and age-friendly physical spaces and resources that foster social connectedness [9]. Current findings suggest that neighborhood perceptions are associated with behavioral and psychosocial factors among NL Black and Latino older adults.

NL Black and Latino older adults share a history of marginalization in the United States, including the Chicago metropolitan area where all participants resided, and it is feasible that comparable factors (e.g., experiences of discrimination and current annual income) may impact their neighborhood perceptions. NL Black and Latino older adults also possess unique trajectories regarding neighborhood environments, including locations of residential areas, nearby resources, and paths of im/migration. These experiences may also contribute to neighborhood perceptions. Indeed, study findings suggest distinct patterns of neighborhood perceptions for NL Black and Latino older adults. For NL Black older adults, neighborhood perceptions were associated with behavioral and psychosocial factors at the interface of structural and social facets. Notably, higher income levels correlated not only to better overall neighborhood perceptions but also more positive views on community cohesiveness and health- and ambient-related aspects of neighborhood environments. Complex and nuanced relationships persist between race, socioeconomic status, and neighborhood characteristics, particularly for Black adults, and it is unclear the precise mechanisms linking higher income to more favorable neighborhood perceptions in this population [47]. It remains crucial to not conflate race and socioeconomic status where racial identification or categorization as Black equates to lower levels of income. Higher income levels may possibly form a foundation for better neighborhood perceptions for NL Black older adults, particularly as an established connection exists between individual financial resources and available residential resources such as grocery stores and green spaces that support positive outcomes in aging. Yet objectively better or presumably more ideal structural aspects of neighborhood environments have not always translated into more optimal outcomes for Black adults [48,49]. For example, more frequent experiences of discrimination that occur near one’s residence may inform poorer perceptions of a neighborhood. A broader group of factors emerged for NL Black older adults regarding community cohesiveness; specifically, with less social isolation, more physical activity, less discrimination, and higher income being associated with more positive perceptions of resident relationships and neighborhood appearance. It is possible that less social isolation, a factor that harkens to positive residential characteristics such as harmony among neighbors [50], is linked to better neighborhood perceptions. Future research is needed to elucidate mechanisms linking these behavioral and psychosocial factors with neighborhood perceptions; however, the current study suggests the existence of a foundational association.

For older Latinos, neighborhood perceptions were associated with behavioral and psychosocial factors centered on communal facets of the self and the environment. Higher purpose in life was the only factor associated with better overall neighborhood perceptions as well as perceived community cohesiveness and ambient surroundings. More late life social activity was also the only factor linked to better perceptions of health opportunities for healthier food selections and physical activities. Hence, the interplay between individual- and group-level factors—rather than either alone—may be instrumental to understanding how people perceive their neighborhood environments [51]. These associations between individual-level factors and neighborhood environments may be connected by a cultural value of familismo, where Latinos highly prioritize connectedness with family and extended social networks, which can serve as a protective factor against deleterious outcomes such as depressive symptoms [52,53,54,55]. Familismo spans across generations and age groups, including older Latinos [54,56]. It is possible that an emphasis on family and social networks among older Latinos shapes how their social capital is cultivated and perceived in neighborhood contexts, including how they conceptualize themselves and their neighborhood-related roles and experiences. Meaning, a sense of community and social integration may foster better neighborhood perceptions among older Latinos. Together, these associations further suggest the intertwined nature of self- and community-related factors as considerations for older Latinos.

While future research is needed to elucidate the directionality of relationships and underlying mechanisms, these findings open a dialogue for targets, such as purpose in life and physical activity, to consider for potential intervention strategies focused on maintaining and improving neighborhood perceptions among NL Black and Latino older adults. Importantly, intervention strategies should exist at the individual, social, and structural levels; specifically, our work suggests that strategies may emphasize income, discrimination, physical activity, late life social activity, and purpose in life, particularly for NL Black and Latino older adults. In varying degrees, these behavioral and psychosocial factors are likely modifiable. For example, strategies focused on individual income may include culturally relevant informational materials and tools regarding financial decision making and choices to protect and maximize current income (individual-level) and can be coupled with national- and state-level efforts regarding pay parity across the lifespan and increased contributions to basic income in older age (structural-level). When combined, these multilevel strategic efforts may benefit structural and social characteristics of neighborhood environments as well as neighborhood perceptions which, in turn, possibly facilitate overall health and quality of life for NL Black and Latino older adults. Intervention strategies focused on structural characteristics of neighborhood environments may also include the development and implementation of available and affordable opportunities for physical activities that are appropriate for a person’s abilities and interests as well as material safety and spaces for engagement in social activities in their own neighborhoods. Intervention strategies may simultaneously target social aspects of neighborhood environments that may foster communal and physical activities through neighborhood-located programming such as group exercise classes or walking groups; thus, creating more opportunities for positive engagement among neighbors and chances for exercise and movement. These efforts may also encourage purpose in life. These levels are likely interrelated, with strategies that focus on any level—individual, social, or structural—having a relationship with other levels and, thus, may maintain or bolster neighborhood perceptions. Overall, perceptions of neighborhood environments represent a critical aspect for NL Black and Latino older adults, but future work remains.

This study has several strengths including a well-characterized group of community-dwelling NL Black and Latino older adults without dementia, the use of established measures of behavioral and psychosocial factors, and a rigorously tested measure of neighborhood perceptions. We also note study limitations. One limitation is the large percentage of women and the relatively smaller number of Latinos, noting a reduced precision for stratified models specific to older Latinos. A second limitation is the cross-sectional design of this study and, as such, it is not possible to determine directionality. Furthermore, other factors not studied here may impact neighborhood perceptions, such as the demographic composition of a neighborhood, segregation, and gentrification. Lastly, we used a binary measure of life space and, thus, limited a fuller understanding of mobility and environmental exposures that may impact neighborhood perceptions.

## 5. Conclusions

The current study set forth associations between behavioral and psychosocial factors and perceptions of neighborhood environments among NL Black and Latino older adults, particularly income for NL Black older adults and purpose in life for older Latinos. Results of this study contribute to the field’s understanding of neighborhood environments as well as another step toward informing needed intervention strategies to benefit health and wellbeing in these populations.

## Data Availability

Data can be requested at https://www.radc.rush.edu (accessed on 30 January 2026).

## Abbreviations

The following abbreviations are used in this manuscript:

NL	Non-Latino
WHO	World Health Organization
RADC	Rush Alzheimer’s Disease Center
MAP	Rush Memory and Aging Project
MARS	Minority Aging Research Study
mPNES	Modified Perceptions of Neighborhood Environments Scale

## Appendix A

**Table A1:** Modified Perception of Neighborhood Environment Scale (mPNES). **Instructions:** I’m going to read you a list of statements about neighborhoods. Please tell me how much you agree or disagree with each statement as it relates to the neighborhood you live in now. In answering these questions, please think of your neighborhood as the area within about a 20-min walk (or about a mile) from your house.

**Item**
Subscale 1: Community Cohesiveness
1. In my neighborhood the buildings and homes are well-maintained. 2. My neighborhood is attractive. 3. It is pleasant to walk in my neighborhood. 4. People around here are willing to help their neighbors. 5. People in my neighborhood generally get along with each other. 6. People in my neighborhood can be trusted.
Subscale 2: Health Opportunities
7. A large selection of fresh fruits and vegetables is available in my neighborhood. 8. There are many opportunities to purchase fast foods in my neighborhood. 9. My neighborhood offers many opportunities to be physically active. 10. Local sports clubs and other facilities in my neighborhood offer many opportunities to get exercise.
Subscale 3: Ambient Surroundings
11. There is a lot of noise in my neighborhood. 12. My neighborhood has heavy traffic.

Response Options:

1—Strongly Agree,

2—Agree,

3—Neutral (neither agree or disagree),

4—Disagree,

5—Strongly Disagree.

**Table A2:** Participant Characteristics * for the Full Sample, Non-Latino Black Older Adults and Older Latinos.

**Variables**	**Full Sample** **(N = 506)**	**Non-Latino Black Older Adults** **(n = 372)**	**Older Latinos** **(n = 134)**
Demographic Characteristics			
Age	79.09 (6.53; 62.56–98.59)	80.66 (6.05; 66.72–98.59)	74.73 (5.83; 62.56–93.11)
Gender, Women	423 (84%)	320 (86%)	103 (77%)
Years of Education	14.26 (4.05; 0.00–30.00)	15.36 (3.13; 5.00–30.00)	11.22 (4.71; 0.00–22.00)
MMSE Score	28.00 (26.00–30.00)	28.00 (26.00–30.00)	28.00 (26.00–29.00)
Outcome: Modified Perceptions of Neighborhood Environments			
Total Scale	47.69 (6.85; 26.00–66.00)	49.65 (6.26; 32.00–66.00)	42.26 (5.32; 26.00–55.00)
Community Cohesiveness Subscale	3.74 (0.55; 1.83–5.00)	3.76 (0.55; 1.83–5.00)	3.70 (0.54; 26.00–55.00)
Health Opportunities Subscale **	2.06 (0.71; −0.25–3.50)	2.04 (0.73; 0.00–3.50)	2.12 (0.65; −0.25–3.50)
Ambient Surroundings Subscale	3.25 (0.78; 1.00–5.00)	3.25 (0.80; 1.00–5.00)	3.25 (0.74; 1.50–5.00)
Behavioral and Psychosocial Factors			
Purpose in Life	3.75 (0.44; 2.00–5.00)	3.83 (0.43; 2.00–5.00)	3.53 (0.38; 2.60–4.60)
Social Isolation	2.33 (0.58; 1.00–4.20)	2.26 (0.55; 1.00–4.20)	2.56 (0.60; 1.00–4.00)
Social Network Size	4.62 (4.48; 0.00–33.00)	4.17 (4.14; 0.00–33.00)	5.84 (5.13; 0.00–28.00)
Late Life Cognitive Activity	2.63 (0.78; 1.00–4.67)	2.74 (0.77; 1.00–4.67)	2.34 (0.73; 1.00–4.33)
Late Life Social Activity	1.74 (0.54; 1.00–3.50)	1.76 (0.54; 1.00–3.50)	1.67 (0.52; 1.00–3.17)
Physical Activity	1.14 (0.92; 0.00–3.00)	1.04 (0.92; 0.00–3.00)	1.44 (0.86; 0.00–3.00)
Life Space	5.00 (5.00–6.00)	5.00 (5.00–6.00)	5.00 (5.00–6.00)
Discrimination	1.67 (2.22; 0.00–9.00)	1.66 (2.35; 0.00–9.00)	1.69 (1.83; 0.00–7.00)
Income	6.43 (2.65; 1–10)	6.82 (2.55; 1–10)	5.33 (2.63; 1–10)

* Mean (Standard Deviation; Range of Scores); Median (Interquartile Range) are reported for the following variables: Income, Mini-Mental State Examination (MMSE), and Life Space; and N (%) for Gender. ** Composite scores included one negatively worded item and the range of composite scores can include a negative integer.

**Table A3:** Relationships between Behavioral and Psychosocial Factors * and Total Scores for the Modified Perceptions of Neighborhood Environments Scale with the Full Sample and Stratified by Racial and Ethnic Groups.

**Factors**	**Full Sample** **(N = 506)**			**Non-Latino Black Older Adults** **(n = 372)**			**Latino Older Adults** **(n = 134)**		
Model 1: Initial Saturated Linear Regression Models									
Adjusted R^2^	0.11			0.14			0.13		
	**Est**	**SE**	***p***	**Est**	**SE**	***p***	**Est**	**SE**	***p***
Purpose in Life	0.07	0.05	0.20	0.02	0.06	0.78	**0.34**	**0.12**	**0.005**
Social Isolation	−0.02	0.04	0.61	−0.02	0.05	0.62	−0.05	0.07	0.51
Social Network Size	0.01	0.00	0.26	0.00	0.01	0.78	0.01	0.01	0.22
Late Life Cognitive Activity	0.04	0.03	0.15	0.03	0.03	0.32	0.05	0.06	0.34
Late Life Social Activity	0.03	0.04	0.49	0.02	0.05	0.70	0.11	0.08	0.19
Physical Activity	***0.06***	***0.02***	***0.005***	***0.06***	***0.03***	***0.01***	0.03	0.04	0.52
Life Space	−0.00	0.02	0.79	−0.00	0.02	0.88	0.00	0.03	1.00
Discrimination	***−0.03***	***0.01***	***0.001***	***−0.03***	***0.01***	***<0.001***	−0.02	0.02	0.43
Income	***0.02***	***0.01***	***0.02***	***0.03***	***0.01***	***0.01***	−0.00	0.02	0.97
Model 2: Final Stepwise Linear Regression Models									
Adjusted R^2^	0.11			0.15			0.13		
	**Est**	**SE**	***p***	**Est**	**SE**	***p***	**Est**	**SE**	***p***
Purpose in Life	***0.10***	***0.05***	***0.04***				***0.44***	***0.10***	***<0.001***
Social Isolation									
Social Network Size							***0.01***	***0.01***	***0.04***
Late Life Cognitive Activity									
Late Life Social Activity									
Physical Activity	***0.07***	***0.02***	***<0.001***	***0.07***	***0.02***	***0.01***			
Life Space									
Discrimination	***−0.03***	***0.01***	***<0.001***	***−0.04***	***0.01***	***<0.001***			
Income	***0.02***	***0.01***	***0.01***	***0.03***	***0.01***	***0.001***			

* Based on multivariable linear regression models controlling for age, gender, and years of education. Statistics provided include parameter estimates, standard errors, and *p*-values. Bold and italicized values indicate statistical significance.

**Table A4:** Relationships between Behavioral and Psychosocial Factors * and Community Cohesiveness Subscale Scores for the Modified Perceptions of Neighborhood Environments Scale with the Full Sample and Stratified by Racial and Ethnic Groups.

**Factors**	**Full Sample** **(N = 506)**			**Non-Latino Black Older Adults** **(n = 372)**			**Latino Older Adults** **(n = 134)**		
**Model 1: Initial Saturated Linear Regression Models**									
Adjusted R^2^	0.10			0.13			0.08		
	**Est**	**SE**	***p***	**Est**	**SE**	***p***	**Est**	**SE**	***p***
Purpose in Life	***0.14***	***0.07***	***0.04***	0.08	0.07	0.30	***0.39***	***0.15***	***0.01***
Social Isolation	−0.04	0.05	0.34	−0.07	0.06	0.21	−0.01	0.09	0.94
Social Network Size	0.01	0.01	0.14	0.01	0.01	0.38	0.01	0.01	0.29
Late Life Cognitive Activity	0.04	0.03	0.23	0.02	0.04	0.59	0.12	0.07	0.11
Late Life Social Activity	0.05	0.05	0.29	0.04	0.06	0.46	0.13	0.10	0.21
Physical Activity	***0.05***	***0.03***	***0.05***	***0.06***	***0.03***	***0.04***	−0.01	0.06	0.59
Life Space	0.00	0.02	0.84	0.01	0.02	0.49	−0.02	0.04	0.89
Discrimination	***−0.03***	***0.01***	***0.004***	***−0.04***	***0.01***	***0.001***	−0.01	0.03	0.60
Income	0.02	0.01	0.07	***0.03***	***0.01***	***0.04***	−0.00	0.02	0.95
**Model 2: Final Stepwise Linear Regression Models**									
Adjusted R^2^	0.10			0.14			0.09		
	**Est**	**SE**	***p***	**Est**	**SE**	***p***	**Est**	**SE**	***p***
Purpose in Life	***0.19***	***0.06***	***<0.001***				***0.48***	***0.13***	***<0.001***
Social Isolation				***−0.11***	***0.05***	***0.02***			
Social Network Size									
Late Life Cognitive Activity									
Late Life Social Activity									
Physical Activity	***0.06***	***0.03***	***0.01***	***0.07***	***0.03***	***0.02***			
Life Space									
Discrimination	***−0.03***	***0.01***	***0.001***	***−0.04***	***0.01***	***<0.001***			
Income	***0.02***	***0.01***	***0.05***	***0.03***	***0.01***	***0.003***			

* Based on multivariable linear regression models controlling for age, gender, and years of education. Statistics provided include parameter estimates, standard errors, and *p*-values. Bold and italicized values indicate statistical significance.

**Table A5:** Relationships between Behavioral and Psychosocial Factors * and Health Opportunities Subscale Scores for the Modified Perceptions of Neighborhood Environments Scale with the Full Sample and Stratified by Racial and Ethnic Groups.

**Factors**	**Full Sample** **(N = 506)**			**Non-Latino Black Older Adults** **(n = 372)**			**Latino Older Adults** **(n = 134)**			
Model 1: Initial Saturated Linear Regression Models										
Adjusted R^2^	0.08			0.10			0.10			
	**Est**	**SE**	***p***	**Est**	**SE**	***p***	**Est**	**SE**	***p***	
Purpose in Life	0.02	0.09	0.84	−0.03	0.10	0.80	0.23	0.18	0.22	
Social Isolation	−0.05	0.06	0.46	−0.07	0.08	0.36	−0.08	0.11	0.47	
Social Network Size	0.01	0.01	0.23	0.00	0.01	0.60	0.01	0.01	0.25	
Late Life Cognitive Activity	0.03	0.05	0.55	0.01	0.05	0.88	0.03	0.09	0.71	
Late Life Social Activity	0.03	0.06	0.66	0.02	0.08	0.84	0.17	0.13	0.18	
Physical Activity	0.07	0.04	0.06	0.06	0.04	0.14	0.06	0.07	0.42	
Life Space	0.01	0.02	0.70	−0.01	0.03	0.60	0.07	0.05	0.15	
Discrimination	−0.02	0.01	0.13	***−0.03***	***0.02***	***0.03***	0.02	0.03	0.59	
Income	0.02	0.01	0.08	***0.05***	***0.02***	***0.005***	−0.02	0.03	0.36	
Model 2: Final Stepwise Linear Regression Models										
Adjusted R^2^	0.08			0.11			0.09			
	**Est**	**SE**	***p***	**Est**	**SE**	***p***	**Est**	**SE**	***p***	
Purpose in Life										
Social Isolation										
Social Network Size										
Late Life Cognitive Activity										
Late Life Social Activity							***0.28***	***0.11***	***0.02***	
Physical Activity	***0.07***	***0.03***	***0.03***							
Life Space										
Discrimination				***−0.03***	***0.02***	***0.02***				
Income	***0.03***	***0.01***	***0.01***	***0.05***	***0.02***	***0.001***				

* Based on multivariable linear regression models controlling for age, gender, and years of education. Statistics provided include parameter estimates, standard errors, and *p*-values. Bold and italicized values indicate statistical significance.

**Table A6:** Relationships between Behavioral and Psychosocial Factors * and Ambient Surroundings Subscale Scores for the Modified Perceptions of Neighborhood Environments Scale with the Full Sample and Stratified by Racial and Ethnic Groups.

**Factors**	**Full Sample** **(N = 506)**			**Non-Latino Black Older Adults** **(n = 372)**			**Latino Older Adults** **(n = 134)**		
Model 1: Initial Saturated Linear Regression Models									
Adjusted R^2^	0.06			0.04			0.14		
	**Est**	**SE**	***p***	**Est**	**SE**	***p***	**Est**	**SE**	***p***
Purpose in Life	***0.19***	***0.10***	***0.05***	0.11	0.12	0.33	***0.62***	***0.20***	***0.002***
Social Isolation	0.01	0.07	0.84	0.04	0.09	0.62	−0.09	0.12	0.42
Social Network Size	−0.00	0.01	1.00	−0.00	0.01	0.88	0.00	0.01	0.94
Late Life Cognitive Activity	0.04	0.05	0.43	0.09	0.06	0.14	−0.14	0.10	0.17
Late Life Social Activity	−0.00	0.07	0.97	−0.02	0.09	0.86	0.01	0.14	0.94
Physical Activity	0.06	0.04	0.12	0.03	0.05	0.54	0.11	0.08	0.15
Life Space	***−0.07***	***0.03***	***0.01***	***−0.07***	***0.03***	***0.04***	−0.05	0.05	0.30
Discrimination	***−0.04***	***0.02***	***0.01***	***−0.04***	***0.02***	***0.04***	−0.04	0.04	0.30
Income	***0.04***	***0.02***	***0.01***	***0.04***	***0.02***	***0.04***	0.04	0.03	0.14
Model 2: Final Stepwise Linear Regression Models									
Adjusted R^2^	0.06			0.03			0.15		
	**Est**	**SE**	***p***	**Est**	**SE**	***p***	**Est**	**SE**	***p***
Purpose in Life	***0.19***	***0.09***	***0.03***				***0.72***	***0.18***	***<0.001***
Social Isolation									
Social Network Size									
Late Life Cognitive Activity									
Late Life Social Activity									
Physical Activity									
Life Space	***−0.07***	***0.03***	***0.01***						
Discrimination	***−0.04***	***0.02***	***0.02***						
Income	***0.05***	***0.02***	***0.001***	***0.05***	***0.02***	***0.01***	***0.06***	***0.03***	***0.03***

* Based on multivariable linear regression models controlling for age, gender, and years of education. Statistics provided include parameter estimates, standard errors, and *p*-values. Bold and italicized values indicate statistical significance.
